# Clinical audit for first-trimester ultrasound screening: a single center experience

**DOI:** 10.3389/fmed.2025.1687183

**Published:** 2025-10-20

**Authors:** Xining Wu, Yixiu Zhang, Hua Meng, Yunshu Ouyang, Zhonghui Xu, Qing Dai, Peipei Zhang

**Affiliations:** Department of Ultrasound, State Key Laboratory of Complex Severe and Rare Diseases, Peking Union Medical College Hospital, Chinese Academy of Medical Sciences and Peking Union Medical College, Beijing, China

**Keywords:** first trimester, quality control, clinical audit, ultrasound, prenatal

## Abstract

**Objective:**

The purpose of this study was to assess the quality of 11–14-week fetal ultrasound images and physician scanning performance in a large general hospital to inform future quality improvement initiatives.

**Methods:**

A retrospective audit of ultrasound scans of normal fetuses at 11–14 weeks was conducted from November 2021 to March 2023 at a large tertiary general hospital in Beijing, China. Ten anatomical views were analyzed by two experienced assessors. Scan completeness and view completeness rates ≥ 70%, and logbook quality scores ≥ 42 (i.e., ≥ 70% of the maximum possible score), were considered acceptable.

**Results:**

The overall scan completeness of 256 logbooks was 77.4%. The scan completeness of 189 logbooks (73.8%) was acceptable. The median image quality score for the 256 logbooks was 37 (interquartile range, 28–46), and 96 logbooks (37.5%) had acceptable image quality, with a score ≥ 42. The scan completeness of 23 sonographers (63.9%) was > 70%. Sonographers with intermediate titles performed a higher average number of fetal ultrasound scans than those with senior titles (148 vs. 115 scans), and their scan completeness and logbook image quality scores were also superior (87% vs. 69% and 43.24 ± 6.38 vs. 31.62 ± 11.28, respectively; both *p* < 0.05).

**Conclusion:**

The majority of sonographers met the expectations of the audit. Sonographers performing more fetal ultrasound scans may have an advantage in terms of scan completeness and image quality.

## Introduction

Performing a routine first-trimester ultrasound examination at 11–14 weeks’ gestation is valuable for confirming viability and plurality, accurate pregnancy dating, screening for aneuploidies, and identifying major structural anomalies (1). A standardized anatomical protocol improves the sensitivity of first-trimester ultrasound screening for fetal anomalies (2). The detection rate for first-trimester screening was 43.1% (3), indicating that approximately half of all fetuses with structural abnormalities detected by prenatal ultrasound can be diagnosed within the first 3 months. This high rate may be attributable not only to the popularization of ultrasound screening training but also to the quality of the ultrasound images obtained.

It is widely believed that for obstetric ultrasound, as part of prenatal screening for fetuses, a quality assurance program should be established. However, quality control studies of prenatal ultrasound have long been based on fetal abnormality detection rates, with few studies paying attention to the scan completeness and standardization of preserved normal views (4). In principle, the archived image quality reflects the overall quality of fetal anatomical screening. During the scanning process, the lack of a robust and continuous monitoring system can lead to variations in the quality of the obtained ultrasound images. Moreover, incomplete or low-quality scanning may lead to uncertainty regarding the effectiveness of ultrasound in detecting fetal abnormalities, which has become a common and serious issue in the field of obstetric ultrasound. Malpractice lawsuits for missed fetal anomalies have become the most common type of litigation involving ultrasound (5). These legal disputes not only cause great harm to patients’ families but also pose a threat to the reputation and normal operation of medical institutions (6).

Regular audits, peer review, and quality assurance procedures improve and sustain good practice (7). For nuchal translucency (NT) and second-trimester ultrasound screening, appropriate feedback and intervention can improve sonographer performance (8–10). Image-based scoring has also proven useful for quality control of fetal biometric and Doppler blood flow measurements (11–13). To achieve optimal results in routine ultrasound examinations, the 11–14-week screening guideline developed by the International Society of Ultrasound in Obstetrics and Gynecology (ISUOG) requires physicians performing early pregnancy scans to participate regularly in established quality assurance programs. However, there have been no reports on clinical audits for first-trimester ultrasound screening of normal fetuses.

Therefore, we designed this study to evaluate the quality of first-trimester fetal ultrasound images acquired in a large general hospital and assess the scanning performance of sonographers through a simple image-based scoring method.

## Materials and methods

### Study design

We conducted a retrospective baseline audit of anonymized routine 11–14 weeks’ gestation fetal scans performed over a 12-month period at a large general hospital with over 3,000 births per annum in Beijing. The systematic scanning of all fetuses was based on the national screening protocol, including two biometric measurements and 10 anatomical-view assessments. Biometric measurements included the crown–rump length (CRL) and NT. There were 10 anatomical views, namely the CRL measurement view, NT view, axial view of fetal head at the level of the lateral ventricles, axial view of fetal thorax at the level of the four-chamber view of the heart, axial view of fetal upper abdomen at the level of the stomach, axial view of fetal abdomen demonstrating the site of umbilical cord insertion, view of bilateral upper limbs, view of bilateral lower limbs, view of placenta demonstrating the site of umbilical cord insertion, and sagittal view of cervix. The screening protocol meets the ISUOG requirements for basic fetal screening at 11–14 weeks, and includes the necessary fetal biometrics and assessment of important anatomical structures. All the sonographers are certified by the regional maternal and child health management organization to perform anatomical evaluations in the first trimester by transabdominal ultrasound. The study was approved by the Research Ethics Committee of Peking Union Medical College Hospital (No. I-22PJ1000); patient informed consent was not required as this study is a retrospective review of routinely collected data.

### Audit process

Ultrasound images and metadata, including the scan date, ultrasound machine used, and sonographer, were extracted from the hospital database (Donghua Ultrasound Imaging Workstation) from November 2021 to March 2023, and the baseline information of the patients was obtained, including age, mode of pregnancy, gestational parity, etc. Cases were limited to singleton pregnancies with no anomalies on the scans. All ultrasound equipment met the national quality requirements.

A total of 36 sonographers carried out the antenatal screening, and the workload varied considerably among them according to the number of hours worked. The total workload of two sonographers was significantly lower than others, with only 12 and 10 cases, respectively. To ensure that the quality of the first-trimester pregnancy ultrasound screening was assessed as comprehensively as possible, we decided to audit all cases from these two sonographers. For the other sonographers, 5% of the cases were selected for audit using simple random sampling. Each logbook, i.e., the image set containing the fetal ultrasound report and all images, was assigned an identification number and anonymized to prevent identification of the assessors.

Prior to initiating the study, two experienced obstetric ultrasound experts (Assessor 1, with > 30 years of experience; and Assessor 2, with > 10 years of experience) validated uniform image criteria and quality scoring protocols to ensure that an independent audit of image completeness and quality could be performed for each logbook.

### Scan completeness

Scan completeness was defined as the proportion of the total number of audited patient scans that were complete (i.e., all required images were obtained). Sonographer scan completeness was defined as the average scan completeness of the audited logbooks. View completeness was defined as the proportion of scans that had at least one image for a particular view.

### Score-based quality assessment

The image quality of the 10 anatomical views was also analyzed. On the basis of scans at 11–14 weeks from ISUOG guideline (1) and taking into account the time-consuming nature of the clinical audit, the two assessors implemented the following simple scoring method by combining anatomical structures and amplification of the views: 3 points, important anatomical structures were clearly displayed and image quality was good; 2 points, most (more than two-thirds) of the important anatomical structures were clearly displayed, although some were poorly displayed, and the image quality was acceptable; 1 point: the view was stored, and the important anatomical structures were visible but poorly displayed; and 0 point, the image quality was insufficient, or no view was kept. The main anatomical landmarks of the 10 views are listed in Table 1. When the sonographer stored multiple views of the same anatomical structure, the view with the highest score was considered in the final score calculation. It should be noted that fetal limbs were scored in the “bilateral upper limbs” and “bilateral lower limbs” views. As an example, if the sonographer stored the “left upper limb” and “right upper limb” views separately, the score for the bilateral upper limbs view was calculated as the average of the scores for the two views. The sum of the scores of two assessors was used as the final score for each logbook, so the maximum possible score for a given logbook was 60 points. Scan completeness and view completeness rates ≥ 70%, and logbook scores ≥ 42 points, were considered acceptable.

**Table 1:** Criteria for quality assessment of ultrasound images in 10 standard views.

	CRL	NT	Head	Thorax	Abdomen	Abdominal wall	Upper limbs	Lower limbs	Placenta	Cervix
View	midsagittal view of fetus	midsagittal view of head and neck	axial view of head	axial view of thoracic	axial view of upper abdomen	axial view of abdomen wall	longitudinal view of bilateral arms	longitudinal view of bilateral legs	longitudinal view of placenta	midsagittal view of cervix
Specific criteria	facial profile, NT and genital tubercle visible	facial profile and NT visible	symmetrical plane with midline and the choroid plexuses visible (butterfly sign)	four-chamber view with two distinct ventricles on grayscale and color Doppler	the fluid-filled stomach in the left	intact anterior abdominal wall and umbilical cord insertion visible	the three segments visible: upper legs, lower legs and feet	the three segments visible: upper arms, lower arms and hands	umbilical cord insertion visible	the internal os visible

CRL, crown–rump length; NT, nuchal translucency.

### Interobserver agreement

To measure agreement, a total of 30 logbooks were selected by a computer randomization program and assessed independently by the two assessors. Interobserver agreement in the quality score for each image was indicated by the kappa statistic.

### Statistical analysis

Scan completeness was expressed as a percentage. The kappa value was calculated to evaluate interobserver agreement, with a kappa value < 0.40 indicating poor agreement, a value of 0.40–0.75 indicating moderate agreement, and a value > 0.75 indicating good agreement. The Kolmogorov–Smirnov test was applied to test the normality of all the data. Mean (standard deviation) values were calculated for normally distributed variables, with medians [interquartile range (IQR)] calculated for variables with skewed distributions. Comparisons between groups of different sonographers were performed using independent-samples t-tests. All analyses (descriptive and comparative) were performed using SPSS version 23 (IBM Corporation, Armonk, NY, United States). All results were considered statistically significant when *p* < 0.05 (two-sided).

## Results

**Figure 1:**
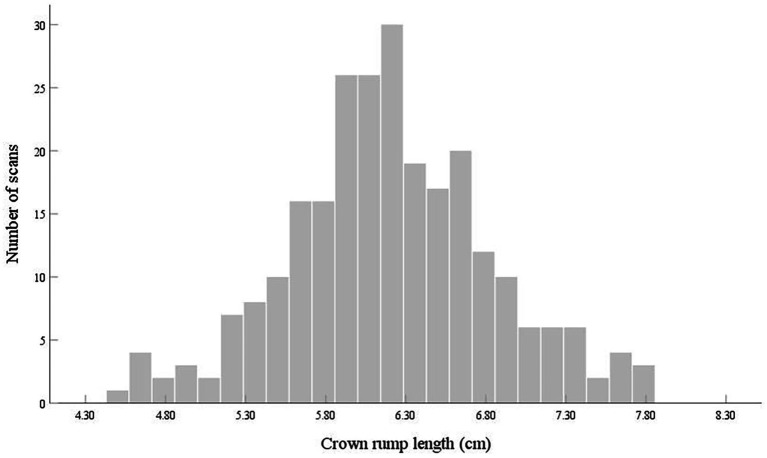
Frequency of fetal first-trimester ultrasound examinations audited in a large tertiary general hospital according to the crown–rump length.

In total, 4,574 logbooks were completed by 36 sonographers. A total of 4,468 images from 256 logbooks were assessed. All scans were acquired at 11–14 weeks, and the mean CRL at the time of the scan was 6.20 ± 0.64 cm (Figure 1). Table 2 shows the characteristics of the study population. All scans were performed using high-end ultrasound equipment, including the Voluson E10 (GE, Boston, MA, United States), Epiq 7 (Philips, Amsterdam, Netherlands), and iU22 (Philips) machines, none of which are calibrated for automatic caliper placement.

**Table 2:** Characteristics of the study population (*N* = 256).

Study population	N (%)
Age (years, mean ± SD)	33 ± 3.5
Mode of pregnancy	
Natural pregnancy	222 (86.7%)
IVF	34 (13.3%)
BMI (Kg/m^2^, median (IQR))	21.3 (19.5–22.5)
Gravidity (median (IQR))	2 (1–2)
Parity (median (IQR))	0 (0–2)
CRL (cm, mean ± SD)	6.20 ± 0.64
Logbooks evaluated for each sonographer (median (IQR))	8 (5–10)
Total number of logbooks submitted per sonographer (median (IQR))	122 (74–182)
Number of images per logbook (median (IQR))	16 (12–21)

BMI, body mass index; CRL, crown–rump length; IQR, interquartile range; IVF, in vitro fertilization.

### Scan completeness and image quality of the logbooks

The overall scan completeness of the 256 logbooks was 77.4%. The scan completeness of 189 logbooks (73.8%) was ≥ 70%, with 63 logbooks (24.6%) having 100% scan completeness. In total, 67 logbooks (26.2%) had < 70% scan completeness, and 39 (15.2%) had ≤ 50% scan completeness.

Completeness varied by view, and the overall scan completeness and completeness in each view is summarized in Table 3. The CRL view completeness was 100%, followed by NT view completeness at 97.3%. For four views, completeness was < 70%: axial view of thorax, axial view of upper abdomen, axial view of abdomen with umbilical cord insertion, and cervical view. The axial view of abdomen with umbilical cord insertion had the lowest completeness (50.8%).

**Table 3:** Overall and individual-view scan completeness of fetal first-trimester ultrasound examinations.

Views	Completeness (%)	Quality score (mean±SD)
CRL	100%	2.50 ± 0.60
NT	97.3%	2.29 ± 0.77
Axial view of the head with the choroid plexuses (butterfly sign)	91.0%	2.59 ± 0.64
Axial view of the thoracic with the four chamber	62.1%	2.84 ± 0.43
Axial view of upper abdomen with stomach	58.6%	2.79 ± 0.47
Axial view of upper abdomen with umbilical cord insertion	50.8%	2.53 ± 0.71
Bilateral upper limbs	88.3%	2.06 ± 0.68
Bilateral lower limbs	85.5%	1.99 ± 0.78
View of placenta with cord insertion	74.6%	2.76 ± 0.55
Midsagittal view of cervix	65.6%	2.76 ± 0.53
Overall	77.4%	2.51 ± 0.31

CRL, crown–rump length; NT, nuchal translucency.

The mean scores of the 10 views are shown in Table 3. The axial view of thorax had the highest image quality, with a mean score of 2.84 ± 0.43. The bilateral lower limbs view had the worst image quality, with a mean score of 1.99 ± 0.78.

### Scan completeness and image quality of the 36 sonographers

The median image quality score for the 256 logbooks was 37 (IQR, 28–46). The image score of 96 logbooks (37.5%) was ≥ 42, and 160 logbooks (62.5%) had a score < 42 (i.e., < 70% of the maximum possible score).

Table 4 shows the scan completeness of the 36 sonographers; 23 sonographers (63.9%) had scan completeness ≥ 70%, but only 2 sonographers (5.6%) had scan completeness of 100%. The scan completeness of 13 sonographers (36.1%) was < 70%. Sonographer number 7 audited two logbooks, for each of which only the NT and CRL views were retained, and the scan completeness was only 20%.

**Table 4:** Scan completeness and image quality scores of first-trimester ultrasound examinations performed by 36 sonographers.

Sonographyer ID	Number of logbooks	Scan completeness (%)	Quality score per logbook (mean ± SD)
1	8	56%	23.44 ± 6.94
2	4	98%	53.75 ± 2.77
3	5	50%	23.20 ± 11.02
4	6	93%	46.17 ± 5.21
5	11	69%	25.55 ± 7.64
6	2	100%	47.25 ± 3.75
7	2	20%	11.00 ± 1.00
8	5	58%	27.80 ± 7.30
9	6	63%	33.17 ± 5.60
10	10	81%	37.05 ± 6.11
11	10	96%	49.70 ± 5.53
12	10	88%	40.00 ± 8.72
13	5	84%	42.60 ± 5.43
14	2	70%	36.50 ± 2.50
15	11	99%	50.00 ± 3.25
16	8	94%	46.38 ± 3.58
17	5	92%	49.90 ± 5.20
18	6	95%	47.83 ± 7.99
19	6	90%	42.83 ± 6.39
20	10	96%	48.35 ± 4.32
21	7	41%	15.57 ± 4.27
22	2	100%	51.50 ± 4.50
23	12	95%	43.17 ± 6.04
24	12	84%	39.58 ± 6.33
25	9	73%	31.67 ± 4.00
26	10	60%	27.55 ± 8.97
27	4	40%	17.25 ± 3.56
28	9	83%	35.44 ± 8.93
29	8	79%	36.94 ± 11.42
30	12	74%	38.33 ± 6.56
31	2	65%	19.50 ± 7.50
32	4	65%	30.00 ± 8.25
33	9	68%	34.22 ± 7.19
34	2	70%	28.50 ± 6.50
35	10	56%	26.45 ± 5.64
36	12	73%	31.21 ± 5.79

Sonographer number 2 had the highest average image quality score (53.75 ± 2.77), whereas sonographer number 7 had the lowest average score (11.00 ± 1.00; difference of 42.75; Table 4).

### Subgroup analysis

Twenty-three sonographers with senior titles had a mean scan number of 115, of whom 13 (56.5%) had a total scan number of > 100, and 13 sonographers with intermediate titles had a mean scan number of 148, of whom 11 (84.6%) had a total scan number > 100. Subgroup analyses were performed for sonographers with different levels of experience and workloads, and the results are shown in Table 5.

**Table 5:** Subgroup analysis of sonographers differing in experience and workload (*N* = 36).

Setting	Sonographers N (%)	Completeness (%)	Quality score (mean ± SD)
Professional qualifications			
Senior title	23 (63.9%)	69%	31.62 ± 11.28
Intermediate title	13 (36.1%)	87%	43.24 ± 6.38
Number of total scans			
≥100	26 (72.2%)	78.3%	37.24 ± 9.77
<100	10 (27.8%)	70.0%	32.96 ± 13.72

The average scan completeness were 69 and 87% for the senior and intermediate sonographers, respectively, and the difference between the two groups was statistically significant (*p* < 0.05). The mean image scores of sonographers with senior and intermediate titles were 31.62 ± 11.28 and 43.24 ± 6.38, respectively, and the difference between the two groups was statistically significant (*p* < 0.05).

There were 24 sonographers with a total workload of more than 100 scans and 12 with a workload of fewer than 100 scans. The former group had a mean scan completeness of 78.3% and a mean image score of 37.24 ± 9.77, which were higher than those of the group with a workload of fewer than 100 scans; the latter group had a scan completeness of 70.0% and a mean image score of 32.96 ± 13.72, although the differences between the two groups were not statistically significant (*p* = 0.236 and *p* = 0.287, respectively).

### Interobserver agreement for image quality

The image quality scores for each view differed slightly between the two assessors (Table 6). The image quality scores for the axial view of upper abdomen had the best agreement, with a kappa value of 1.000. The scores for the CRL view had the lowest agreement, with a kappa value of 0.764.

**Table 6:** Interobserver agreement in quality scores for 10 views (up to 30 logbooks per view).

Views	Number	Kappa value	95% Confidence interval
CRL	30	0.764	0.433–0.943
NT	30	0.802	0.490–0.886
Axial view of head with the choroid plexuses (butterfly sign)	28	0.913	0.519–0.857
Axial view of thoracic with the four chamber	24	0.784	0.280–1.096
Axial view of upper abdomen with stomach	25	1.000	0.688–0.688
Axial view of upper abdomen with umbilical cord insertion	22	0.900	0.496–0.880
Bilateral upper limbs	27	0.866	0.508–0.868
Bilateral lower limbs	28	0.942	0.578–0.798
View of placenta with cord insertion	26	0.824	0.467–0.909
Midsagittal view of cervix	25	0.840	0.382–0.994

CRL, crown–rump length; NT, nuchal translucency.

## Discussion

In this audit, we assessed the first-trimester scanning performance of sonographers, including in terms of scan completeness and image quality. To our knowledge, this is the first relatively large-scale clinical audit of first-trimester ultrasound screening conducted in a Chinese hospital. International guideline (1) provide prescriptive guidance on first-trimester ultrasound screening views and specific requirements but, in practice, there are some variations in view requirements between countries, or even between regions within a country, attributable to local policy and skill level differences.

Proper storage of prenatal ultrasound images can help the sonographer avoid and defend against litigation (14). In clinical practice, regulatory authorities worldwide impose requirements on sonographers, such as participation in continuing medical education activities and performance of a minimum number of scans, but evaluation of scan quality during the course of the sonographer’s actual work remains insufficient (15). This insufficiency is attributable to the need for manual audits by experts, which are subjective and time-consuming. Furthermore, the degree of implementation of screening protocols is not consistent among sonographers, leading to variations in the quantity and quality of stored clinical scans. The majority of the sonographers in this study were able to meet the quality requirements of the screening protocol in their practice, with a scan completeness of 77.4%. However, 15.2% of logbooks were missing approximately half of the images required by the screening program. Failure to retain complete images may mean that certain anatomical structures are missed during scanning, which leads to a much higher risk of missing fetal structural abnormalities and a potential risk of litigation.

In the audited logbooks, the CRL view had the highest scan completeness (100%). However, surprisingly, scan completeness for the NT view was not 100%, which may call into question the accuracy of NT measurements, where good practice in fetal biometry is likely correlated with good practice in anatomical screening. Fetal biometry is determined using standardized ultrasound planes, contributing to the accuracy of the measurements and reducing interobserver variability (16). Reviewing the seven cases without NT views, it was evident from the images that the fetal position was not suitable for obtaining a satisfactory view for NT measurement; furthermore, the high intensity of the work resulted in the sonographer not having sufficient time. Scan completeness was low in the axial view of fetal thorax and upper abdomen, and in the axial view of abdomen demonstrating the site of umbilical cord insertion (50.8%). These axial views are important for assessing the integrity of the thoracic and abdominal wall. Syngelaki et al. performed standardized early pregnancy ultrasound screening on more than 100,000 fetuses, and the detection rate of thoracic and abdominal wall defects such as pentalogy of Cantrell, ectopia cordis, and gastroschisis was 100% (17). Liao et al. showed that standardized early pregnancy scans detected 95.6% of abdominal wall defects (3). Moreover, scan completeness of cervical view was low, probably because the sonographers’ attention was more focused on screening fetal structures, neglecting scanning of the cervix.

Anatomical landmarks are the basis for reliable and detailed screening of fetal structures (18). Although we used a simplified image quality scoring system, the quality evaluation was still valid and practical. The median image score of the 256 logbooks was 37, and the percentage of logbooks with scores ≥ 42 was only 37.5%. The lowest scores were those for the view of bilateral lower limbs. According to the guideline (1), bilateral lower limb views are required to show three limb segments during the scanning process, however, the sonographer may store separate views of the right and left limbs or limb segments because of the fetal position or fetal movement. This approach does not strictly follow the guidelines, and the same problem exists in the upper limb views.

Regular clinical audits can also identify sonographers who need targeted feedback and retraining to improve their ability to obtain standardized views and accurately identify fetal anatomy (19, 20). For example, sonographer number 7 had a scan completeness of only 20%, well below the guideline rate. Sonographer number 8 audited five logbooks and failed to store NT views in four cases. In addition, we found that intermediate-title sonographers had a higher fetal ultrasound examination workload, as well as higher scan completeness and image quality, than senior sonographers, suggesting that workload is related to proficiency and that an accumulation of work is the basis for achieving standardized scans and improving quality.

Interobserver agreement in image scores was good. Agreement was highest for the axial view of upper abdomen because the stomach is usually visible at this stage, and it is easy to standardize the view. The lowest agreement was observed for CRL view. The CRL measurement requires accurate magnification of the image, a midsagittal fetus position, a horizontal fetus position, accurate placement of the calipers, and amniotic fluid that can be visualized below the chin of the fetus (19). In contrast, our simple scoring method may lead to discrepant results because of insufficiently refined criteria. Previous studies have shown that the fetal midsagittal position has the greatest impact on CRL measurement (21), and scoring consistency may be improved if the scoring criteria include specific details or weights.

This study has several strengths. We audited scans in real work situations, and the sample images were randomly selected from among pregnancies that were routinely screened at 11–14 weeks. Through our method of assessing scan completeness, important views that might be missed can be identified. Additionally, good practice in clinical audits of images in early pregnancy may allow more planes and anatomical structures to be displayed in mid-pregnancy. In our study, image quality assessment was performed by experienced obstetric ultrasound experts, which is a reliable way of auditing. Although the image quality scoring method was simple, it had already demonstrated its effectiveness and practicality. The clinical audit experiences mentioned above can serve as a reference or validation for other medical institutions enabling them to regularly assess the technical proficiency of their sonographers and develop well-informed training programs.

There are several shortcomings to this study. A limited number of selective audits were performed by each sonographer, which may not provide adequate insight into their performance. Due to the limitations of the retrospective study and the low incidence of congenital anomalies, we were unable to analyze the relationship among image quality, completeness, and the actual anomaly detection rate; therefore, the results do not fully reflect the ability of clinical diagnoses. Moreover, the quality evaluation of first-trimester ultrasound approaches primarily originates from studies conducted in single tertiary care facilities, which restricts the generalizability of the results of this study to the broader population. Our comprehensive manual audit is very labor intensive for a high-intensity clinical setting, which would be a major barrier to implementation in routine practice.

However, despite these limitations, our retrospective study has still provided valuable insights. It enabled us to identify trends and patterns in scan quality and completeness among a significant number of cases within the available data. These preliminary findings can lay the foundation for future multi-center prospective studies. In future studies, large-scale, multi-center studies can be carried out, involving numerous hospitals with varying equipment levels and sonographers with different professional skills. Artificial intelligence and machine learning technologies can be introduced to assess scan completeness and image quality by recognizing key anatomical views (22). By combining the results of image evaluation with the actual anomaly detection rate, a further analysis of its impact on the anomaly detection rate can be conducted.

## Conclusion

In conclusion, most sonographers met the expectations of the audit. Storage of axial views of fetal thorax and abdomen, the cervical view, and standardization of bilateral lower limb views are areas for future attention. Although clinical audits are time consuming, they remain important for fetal ultrasound screening quality assessment. This type of quality assessment can help departments better understand the performance of sonographers and analyze and summarize findings for continuous quality improvement. Improved sonographer skills can facilitate identify certain fetal abnormalities that require clinical intervention or prenatal decision-making, thus allowing patients to benefit from early diagnosis. Sonographers with incomplete view storage and poor-quality images should be better trained, and it is recommended that the examination time be adequate to ensure a sufficient number of high-quality images are obtained.

## Data Availability

The original contributions presented in the study are included in the article/supplementary material, further inquiries can be directed to the corresponding authors.
